# Arthroscopic Lateral Tibial Plateau Translation Correlates With Pivot‐Shift Severity in Anterior Cruciate Ligament‐Deficient Knees

**DOI:** 10.1002/ars2.70113

**Published:** 2026-10-22

**Authors:** Horacio Rivarola, Camilo Helito, Cristian Collazo, Marcos Palanconi, Marcos Meninato, Carlos Peñaherrera‐Carrillo, Alejandro Barros Castro, Bautista Rivarola, Francisco Endara Urresta

**Affiliations:** ^1^ Department of Orthopaedics and Traumatology Hospital Universitario Austral Buenos Aires Argentina; ^2^ Department of Orthopaedics University of São Paulo São Paulo Brazil; ^3^ Hospital Vozandes Quito Ecuador; ^4^ Universidad Internacional del Ecuador Quito Ecuador; ^5^ Hospital Universitario Austral Buenos Aires Argentina; ^6^ Department of Orthopaedics and Traumatology Clinica Arthros Quito Ecuador

## Abstract

**Purpose:**

To quantify arthroscopic lateral tibial plateau (LTP) translation in anterior cruciate ligament (ACL)‐deficient knees using a standardized intraoperative protocol and to evaluate its association with pivot‐shift grade and lateral tibial slope, including its ability to discriminate high‐grade pivot‐shift.

**Methods:**

Patients aged 18 to 45 years with an isolated ACL rupture confirmed by clinical examination, magnetic resonance imaging, and arthroscopy who met the predefined eligibility criteria and had arthroscopic video of adequate quality for measurement underwent standardized arthroscopic assessment of LTP translation. Arthroscopic LTP translation was quantified from standardized high‐definition arthroscopic video recordings using a calibrated probe during valgus and internal rotation stress at 30° of knee flexion. Measurements were independently repeated by 2 observers. Associations with preoperative pivot‐shift grade and lateral tibial slope were assessed. Diagnostic performance was evaluated using receiver operating characteristic analysis.

**Results:**

Sixty patients (38 men, 22 women; mean age 29.8 ± 6.7 years) were included in the final analysis. Mean arthroscopic LTP translation was 4.6 ± 1.7 mm (range, 2‐8 mm). Reproducibility was excellent (intraclass correlation coefficient intra = 0.91; inter = 0.89). LTP translation strongly correlated with pivot‐shift grade (*r* = 0.72, *P* < .001) and moderately with lateral tibial slope (*r* = 0.51, *P* = .003). Receiver operating characteristic analysis showed an area under the curve of 0.85 (95% confidence interval, 0.77‐0.93). A threshold of >5 mm yielded 82% sensitivity and 78% specificity for detecting high‐grade pivot‐shift.

**Conclusions:**

Arthroscopic quantification of LTP translation is a reproducible and clinically relevant method for objectively assessing rotational instability in ACL‐deficient knees. Arthroscopic quantification of LTP translation showed excellent inter‐ and intraobserver reliability and was strongly associated with preoperative pivot‐shift grade. A translation threshold greater than 5 mm showed good discriminative performance for identifying high‐grade pivot‐shift.

**Level of Evidence:**

Level III, retrospective comparative study.

## Graphical abstract

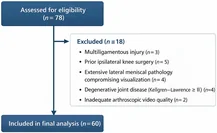

Rotational instability remains one of the most challenging and incompletely resolved problems in anterior cruciate ligament (ACL) surgery. Despite advances in graft selection, tunnel positioning, and fixation techniques, residual pivot‐shift persists in a substantial proportion of patients and continues to be associated with inferior functional outcomes and graft overload.^1^, ^2^, ^3^, ^4^ The pivot‐shift phenomenon—characterized by coupled anterior translation and internal rotation of the lateral tibial plateau (LTP) during dynamic knee motion—remains among the most sensitive clinical indicators of functional instability. However, its assessment is inherently subjective and influenced by examiner technique, patient relaxation, and testing conditions, resulting in inconsistent reproducibility even when standardized grading systems or adjunctive devices are used.^5^, ^6^, ^7^, ^8^, ^9^, ^10^

Although extensive biomechanical and imaging research has sought to clarify the mechanisms underlying rotational instability, limited attention has been directed toward its direct arthroscopic quantification. During diagnostic arthroscopy, excessive mobility of the LTP in ACL‐deficient knees can be readily visualized, particularly under combined valgus and internal rotation stress.^1^, ^2^, ^3^, ^11^, ^12^, ^13^ Despite this consistent intraoperative observation, it has remained largely qualitative, and traditional arthroscopic descriptions have not translated this visual phenomenon into a reproducible numerical measurement.^3^, ^4^, ^11^, ^14^, ^15^, ^16^, ^17^ As a result, a gap persists between surgical perception and objective biomechanical characterization.^3^, ^4^, ^11^, ^14^, ^15^

The absence of a quantitative intraoperative parameter has practical implications for surgical decision‐making, especially regarding the indication for anterolateral reinforcement procedures such as lateral extra‐articular tenodesis or anterolateral ligament reconstruction.^1^, ^2^, ^3^, ^4^ Although these adjunctive techniques are known to reduce rotational laxity, their use is still primarily guided by subjective clinical assessment rather than measurable intraoperative thresholds.^4^, ^11^, ^14^ An objective arthroscopic marker could therefore provide real‐time, evidence‐based support for tailoring reconstruction strategies to the individual degree of rotational instability.^16^, ^17^

In this context, arthroscopic LTP translation—defined as the measurable displacement of the LTP relative to the femoral condyle under standardized rotational stress—represents a potentially quantifiable manifestation of rotational laxity.^12^, ^13^, ^18^, ^19^ Measurement of this translation in millimeters using a calibrated arthroscopic probe may allow transformation of a subjective intraoperative observation into an objective and reproducible metric.^5^, ^7^, ^8^

The purpose of this study was to quantify arthroscopic LTP translation in ACL‐deficient knees using a standardized intraoperative protocol and to evaluate its association with pivot‐shift grade and lateral tibial slope (LTS), including its ability to discriminate high‐grade pivot‐shift. We hypothesized that arthroscopic LTP translation represents an objective surrogate marker of rotational instability, correlates with clinical pivot‐shift grade, and discriminates high‐grade pivot‐shift with clinically useful diagnostic accuracy.

## METHODS

### Study Design

This study was designed as a retrospective observational investigation focused on quantifying dynamic arthroscopic behavior of the LTP in ACL‐deficient knees. All aspects of design, data collection, and reporting followed the Strengthening the Reporting of Observational Studies in Epidemiology guidelines to ensure methodological transparency and reproducibility.

### Patient Selection

The study was carried out at a single tertiary‐level, high‐volume sports medicine center with extensive experience in arthroscopic knee reconstruction. The research aimed to (1) develop a standardized intraoperative method to measure LTP translation under rotational stress and (2) correlate this measurement with preoperative clinical and imaging markers of rotational instability.

Between January 2023 and April 2025, patients presenting with clinically and radiologically confirmed ACL rupture were screened for eligibility. Inclusion criteria were age 18 to 45 years, isolated ACL rupture without associated ligament injury, and availability of high‐quality arthroscopic video recordings allowing clear visualization of the LTP‐femoral condyle interface. Exclusion criteria were multiligamentous injury (medial collateral ligament, posterior cruciate ligament, posterolateral corner, or anterolateral ligament), previous ipsilateral knee surgery, irreparable or extensive lateral meniscal pathology or prior lateral meniscectomy compromising visualization, degenerative joint disease (Kellgren‐Lawrence ≥ II) or bony deformity affecting tibiofemoral congruence, and inadequate video quality for measurement. Patient identification, eligibility assessment, exclusions, and final enrollment are summarized in Figure 1.

**FIGURE 1:**
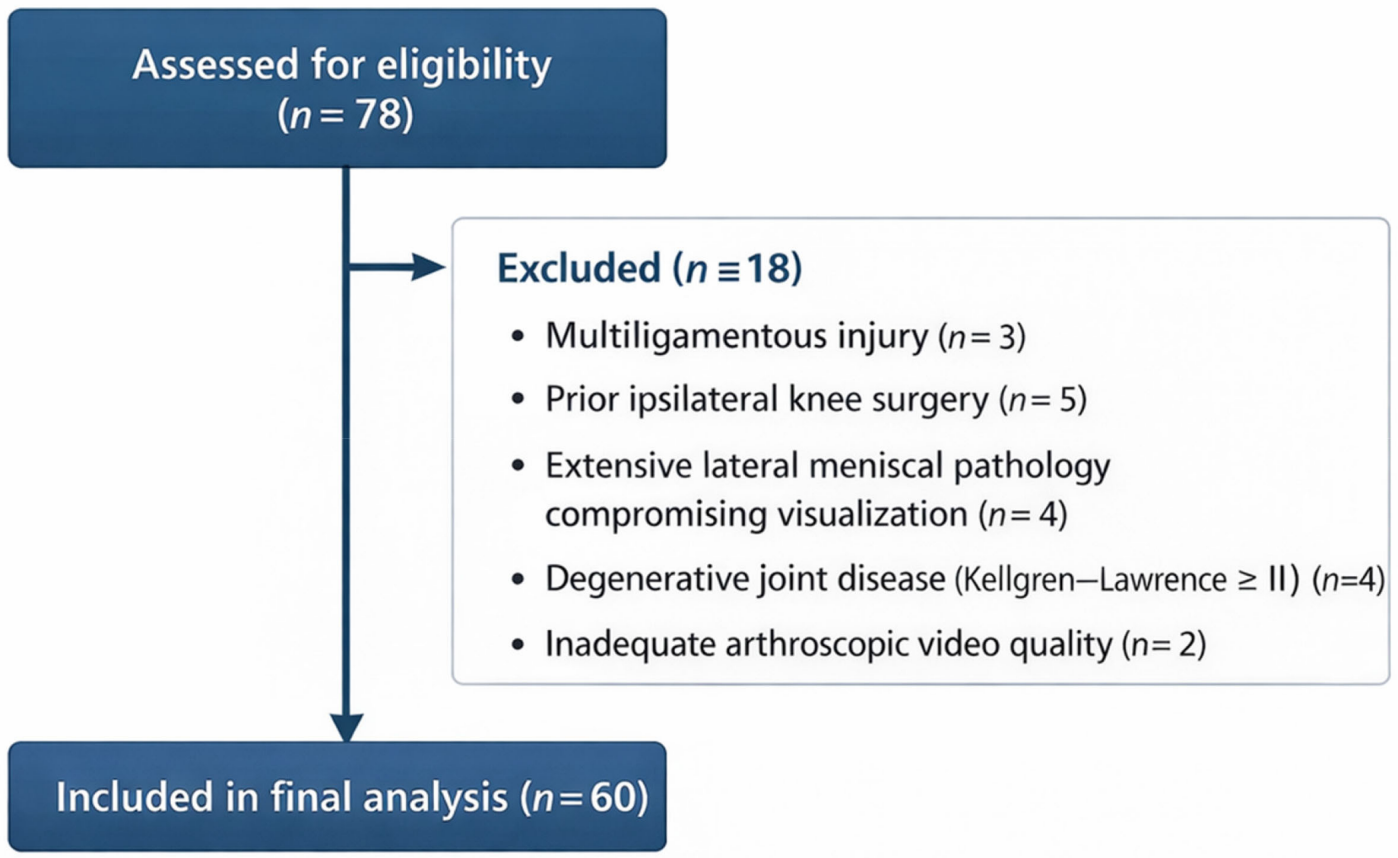
Patient inclusion and exclusion flowchart. Flow diagram illustrating patient screening, exclusion based on predefined criteria, and final cohort included in the analysis. Patients were excluded because of multiligamentous injury, prior ipsilateral knee surgery, extensive lateral meniscal pathology compromising visualization, degenerative joint disease (Kellgren‐Lawrence grade ≥ II), or inadequate arthroscopic video quality.

Ethical approval was obtained from the Institutional Review Board of Clínica Arthros (ARTC‐0030). All participants provided written informed consent for study participation and for the use of anonymized arthroscopic recordings for research and publication purposes.

Patient confidentiality was protected throughout data handling and analysis, with all identifying information removed before video analysis. No financial incentives were offered, and all investigators declared the absence of conflicts of interest related to the study. All procedures were performed by 3 senior surgeons (H.R., C.C., M.M.) adhering to a unified operative and video documentation protocol.

### Arthroscopic Setup

Arthroscopic procedures were performed under spinal or general anesthesia, with patients positioned supine and the operative leg stabilized using a lateral thigh post and a foot roll. The knee was positioned at approximately 30° of flexion at the start of each measurement, with brief controlled motion allowed only to apply valgus and internal rotation stress during assessment.

Both spinal and general anesthesia were used according to anesthesiology preference and patient‐specific factors. Because all measurements were performed after complete muscle relaxation, anesthetic technique was not expected to influence the magnitude of arthroscopic translation and was therefore not considered a confounding variable. Visualization of the lateral compartment was performed through the anteromedial portal, whereas the calibrated probe was introduced through the anterolateral portal to allow direct access to the posterior margin of the LTP.

Each procedure was recorded using a high‐definition arthroscopic camera system (1080p resolution, 30 frames per second) integrated with a spatial calibration grid placed within the optical field at the start of the procedure. A calibrated reference grid was briefly introduced into the arthroscopic field at the beginning of each procedure to verify correspondence between the probe's millimetric markings and the optical magnification of the arthroscopic system. The grid was removed prior to measurement and was used solely for calibration purposes.

### Measurement Protocol

After standard diagnostic evaluation, dynamic assessment of LTP translation was performed prior to ACL reconstruction. A calibrated arthroscopic probe marked at 1‐mm increments was used as the measurement instrument.

With the knee maintained at 30° of flexion, the probe tip was placed against the posterior margin of the LTP, directly below the posterior horn of the lateral meniscus. The assistant surgeon (F.E.U.) then applied a standardized maneuver combining valgus stress and internal tibial rotation, replicating the clinical pivot‐shift. Under direct visualization, the anterolateral displacement of the LTP relative to the lateral femoral condyle was observed and recorded.

The maximal translation—defined as the greatest measurable excursion of the tibial plateau during the maneuver—was quantified in millimeters by aligning the probe markings with fixed anatomical references, including the posterior cortical margin of the lateral femoral condyle and the adjacent subchondral contour of the LTP. Each measurement was recorded on video from the same optical angle to minimize parallax error.

All recordings were independently analyzed by 2 experienced arthroscopic surgeons (H.R., M.M.) who were blinded to clinical and magnetic resonance imaging (MRI) data. Each observer performed 2 independent measurements for every case, separated by at least 2 weeks, to assess intraobserver repeatability. When discrepancies greater than 1 mm were noted, a third blinded reviewer (A.B.C., B.R.) re‐evaluated the video and provided a consensus value. The mean of all validated measurements was used for subsequent statistical analysis. Independent intraoperative measurements performed by different surgeons (C.C., M.P., C.P‐C., F.E.U.) on the same knee were considered but were not feasible within the operative workflow and study design.

### MRI Evaluation

All patients underwent preoperative 1.5‐T MRI (Siemens Magnetom, Erlangen, Germany) with sagittal T2‐weighted sequences (slice thickness 3 mm, field of view 160 mm). The LTS was determined according to a validated geometric protocol. The tibial anatomical axis was defined by connecting the midpoints of the tibial diaphysis at 5 and 15 cm distal to the joint line. A line tangent to the subchondral surface of the LTP was then drawn. The LTS was measured as the acute angle between this tangent and a line perpendicular to the tibial anatomical axis. All MRI measurements were performed twice by a fellowship‐trained musculoskeletal radiologist (F.R.) blinded to arthroscopic and clinical data, and the mean value was used for analysis.

### Statistical Analysis

Data were analyzed using R statistical software (version 4.3.1, R Foundation for Statistical Computing, Vienna, Austria). Normality of continuous variables was verified with the Shapiro‐Wilk test.

Intra‐ and interobserver reliability analyses were performed to assess the reproducibility of measurements obtained from standardized arthroscopic video recordings rather than real‐time intraoperative measurements. LTP translation measurements were assessed using the intraclass correlation coefficient (ICC) under a 2‐way random‐effects model for absolute agreement. ICC values >0.75 were considered excellent.

Pearson's correlation coefficients (*r*) were used to evaluate relations between LTP translation, clinical pivot‐shift grade (graded 0‐3 according to the International Knee Documentation Committee), and LTS (in degrees).

Receiver operating characteristic curves were constructed to determine the ability of arthroscopic LTP translation to predict a high‐grade pivot‐shift (grade ≥ 2). The area under the curve, optimal cutoff point (Youden index), sensitivity, and specificity were calculated.

A binary logistic regression model was used to adjust for potential confounders such as age, sex, and LTS in predicting high‐grade pivot‐shift. All statistical tests were 2‐tailed, and significance was set at *P* < .05.

Sex‐stratified exploratory analyses were additionally performed to evaluate potential effect modification, although the study was not powered to detect sex‐specific differences. LTP translation was initially analyzed as a continuous variable to evaluate its correlation with pivot‐shift grade across the full spectrum of instability. Dichotomization at pivot‐shift grade ≥ 2 was subsequently performed for diagnostic and clinical interpretability, because this threshold is commonly used to define clinically relevant rotational instability and to guide consideration of adjunctive procedures.

## RESULTS

Of the 78 screened knees, 18 were excluded because of failure to meet inclusion criteria, including multiligamentous injury, previous ipsilateral knee surgery, extensive lateral meniscal pathology compromising visualization, degenerative joint disease (Kellgren‐Lawrence ≥ II), or inadequate arthroscopic video quality. A total of 60 ACL‐deficient knees from 60 consecutive patients were included in the final analysis. The mean age of the cohort was 29.8 ± 6.7 years (range, 18‐44), comprising 38 men (63.3%) and 22 women (36.7%). The mean body mass index was 26.9 ± 3.8, and the mean interval from injury to surgery was 4.1 ± 2.3 months. Most patients (68%) participated in pivoting sports, including soccer (n = 27), basketball (n = 7), rugby (n = 5), and volleyball (n = 2). Twelve patients (20%) sustained injuries during recreational athletic activities and 7 (12%) during nonsporting incidents such as falls or traffic accidents. All procedures were completed without intraoperative complications, and arthroscopic video quality was sufficient for analysis in all cases. There were no differences in demographic or baseline characteristics (*P* > .05 for all comparisons). Baseline demographic and clinical characteristics are summarized in Table 1.

**TABLE 1:** Baseline Demographic and Clinical Characteristics of the Cohort

**Characteristic**	**Value**
Age, yr	28.8 ± 6.0
Male sex, n (%)	37 (61.7%)
BMI	27.1 ± 3.8
Time from injury to surgery, mo	4.4 ± 2.3
Participation in pivoting sports, n (%)	47 (78.3%)

*Note*: Continuous data are presented as mean ± standard deviation and categorical data as count and percentage.

BMI, body mass index; mo, months; yr, years.

The reproducibility analysis showed excellent inter‐ and intraobserver agreement for measurements obtained from standardized arthroscopic video recordings. The intraobserver reliability reached an ICC of 0.91 (95% confidence interval [CI], 0.87‐0.95), and interobserver reliability yielded an ICC of 0.89 (95% CI, 0.84‐0.94).

The mean absolute difference between repeated measurements by the same observer was 0.4 ± 0.3 mm, indicating minimal intraobserver variability. These results confirm that the measurement technique is both consistent and reproducible across observers and repeated evaluations (Table 2).

**TABLE 2:** Intraobserver and Interobserver Reliability of Arthroscopic LTP Translation Measurements

**Reliability Metric**	**Point Estimate**	**95% CI**	**Model**
Intraobserver ICC	0.91	0.87‐0.95	Two‐way random, absolute agreement
Interobserver ICC	0.89	0.84‐0.94	Two‐way random, absolute agreement

*Note*: ICCs were calculated using a 2‐way random‐effects model (absolute agreement). Both intraobserver and interobserver reliability showed excellent consistency across repeated evaluations.

CI, confidence interval; ICC, intraclass correlation coefficient; LTP, lateral tibial plateau.

The mean arthroscopic LTP translation was 4.6 ± 1.7 mm, with a range from 2 to 8 mm. Distribution analysis showed a near‐normal curve (Shapiro‐Wilk *P* = .13). When stratified by sex, mean arthroscopic LTP translation was 4.7 ± 1.6 mm in male patients and 4.4 ± 1.8 mm in female patients, with no statistically significant difference between groups (*P* = .36). The correlation between arthroscopic translation and pivot‐shift grade remained significant in both men (*r* = 0.70, *P* < .001) and women (*r* = 0.73, *P* < .001).

Patients engaged in pivoting sports exhibited slightly greater mean translation (4.9 ± 1.6 mm) compared with nonathletes (4.1 ± 1.5 mm), although this difference did not reach statistical significance (*P* = .08). The intraoperative visualization consistently revealed a characteristic anterior displacement of the LTP under applied valgus and internal rotation, followed by a sudden reduction on the release of stress—corresponding to the visually observed anterolateral translation of the LTP under applied rotational stress (Figure 2).

**FIGURE 2:**
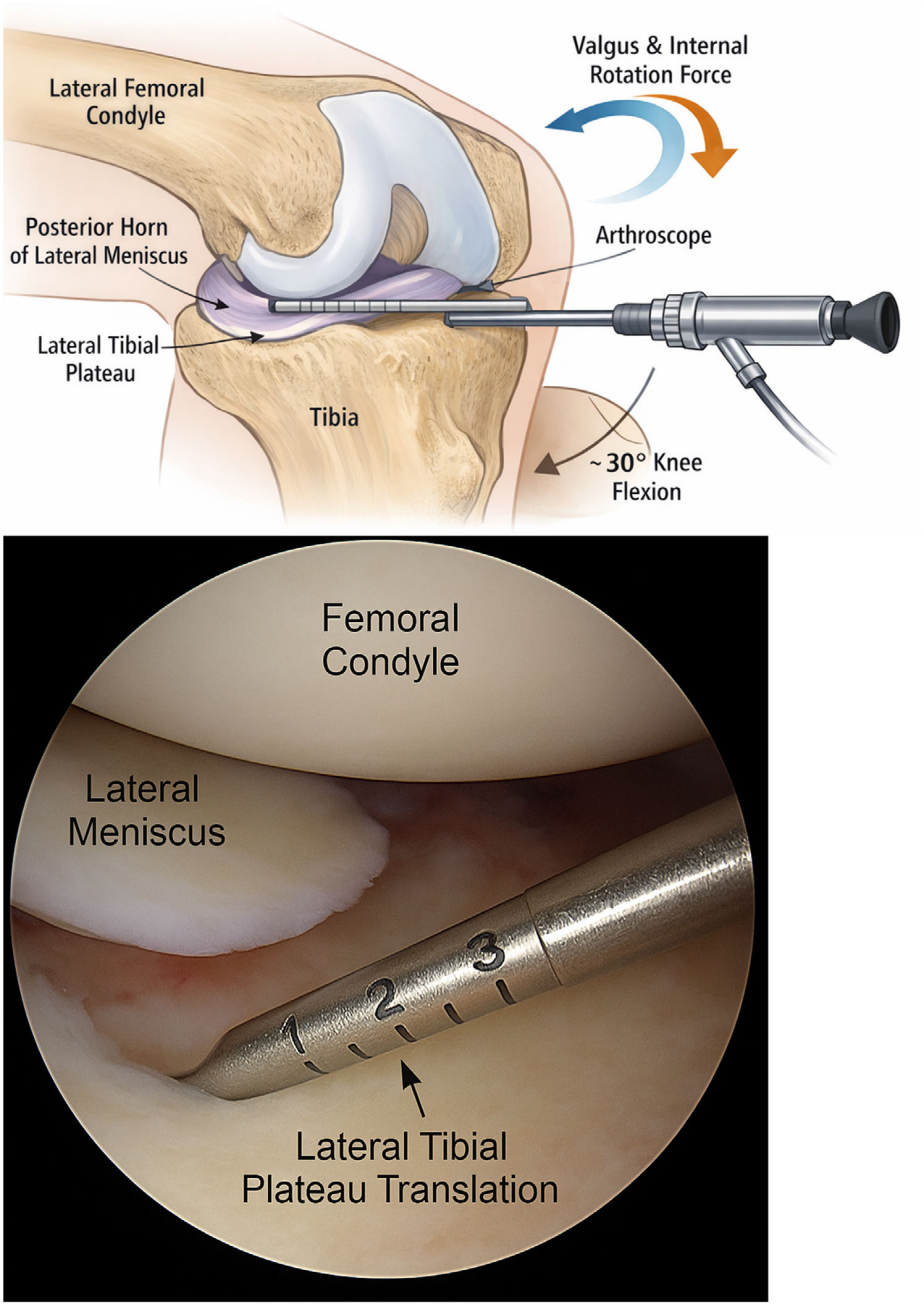
Arthroscopic schematic illustrating measurement of LTP translation using a calibrated 1‐mm probe at 30° of knee flexion. The probe is positioned against the posterior edge of the LTP beneath the posterior horn of the lateral meniscus. With the knee flexed at 30°, a combined valgus and internal rotation stress is applied, producing anterolateral translation of the tibial plateau relative to the femoral condyle. The maximal excursion, measured directly on the probe's 1‐mm markings, represents the arthroscopic quantification of the “hidden pivot.” (LTP, lateral tibial plateau.)

A strong positive correlation was observed between arthroscopic LTP translation and clinical pivot‐shift grade (*r* = 0.72, *P* < .001), indicating that greater translation was associated with higher grades of clinical instability. Additionally, a moderate correlation was found between LTP translation and LTS measured on preoperative MRI (*r* = 0.51, *P* = .003), suggesting that increased posterior slope may contribute to greater anterolateral mobility of the tibial plateau (Tables 3 and 4). No significant associations were observed between LTP translation and demographic factors such as age (*r* = 0.12, *P* = .34) or body mass index (*P* = .09, *P* = .41) (Figure 3).

**TABLE 3:** Correlation Coefficients Between Arthroscopic LTP Translation, Clinical Pivot‐Shift Grade, and Lateral Tibial Slope

**Correlation**	***r***	***P*** **Value**
LTP translation versus pivot‐shift grade	0.71	<.001
LTP translation versus lateral tibial slope, °	0.55	<.3

*Note*: Pearson correlation coefficients (*r*) with corresponding *P* values are shown. A strong association was found between LTP translation and pivot‐shift grade and a moderate association with lateral tibial slope.

LTP, lateral tibial plateau.

**TABLE 4:** Multivariate Logistic Regression Model Identifying Independent Predictors of High‐Grade Pivot‐Shift (Grade ≥ 2)

**Predictor**	**OR**	**95% CI**	***P*** **Value**
LTP translation >5 mm	3.40	1.60‐7.10	<.2
Lateral tibial slope (per degree)	1.28	1.05‐1.63	.10
Age (per yr)	0.99	0.95‐1.03	.62
Male sex	1.12	0.58‐2.21	.73

*Note*: OR with 95% CIs and *P* values are reported. LTP translation greater than 5 mm remained an independent predictor of high‐grade pivot‐shift after adjusting for lateral tibial slope, age, and sex.

CI, confidence interval; LTP, lateral tibial plateau; OR, odds ratio; yr, year.

**FIGURE 3:**
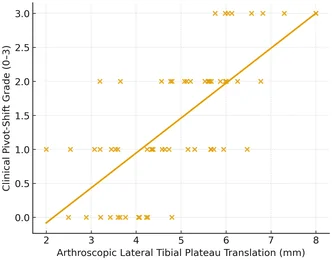
Scatter plot showing the correlation between arthroscopic LTP translation and clinical pivot‐shift grade (*r* = 0.72, *P* < .001). Each dot represents 1 ACL‐deficient knee (n = 60). A strong positive relation is observed between the magnitude of intraoperative LTP translation and the preoperative clinical pivot‐shift grade (0‐3). The solid line represents the least‐squares regression fit, illustrating the progressive increase in measured translation with higher instability grades. (ACL, anterior cruciate ligament; LTP, lateral tibial plateau.)

Receiver operating characteristic analysis showed that arthroscopic LTP translation effectively predicted a high‐grade pivot‐shift (grade ≥ 2). The area under the curve was 0.85 (95% CI, 0.77‐0.93), indicating excellent discriminative ability. A cutoff value of >5 mm yielded the optimal diagnostic balance with 82% sensitivity and 78% specificity for identifying knees with high‐grade pivot‐shift. At this threshold, the positive predictive value was 0.81, and the negative predictive value was 0.79.

In the multivariate logistic regression model, LTP translation >5 mm remained a significant independent predictor of high‐grade pivot‐shift (odds ratio = 3.4, 95% CI 1.6‐7.1; *P* = .002) after adjusting for tibial slope, age, sex, and body mass index. LTS also emerged as a secondary predictor (odds ratio = 1.28 per degree, 95% CI 1.05‐1.63; *P* = .01) (Figure 4).

**FIGURE 4:**
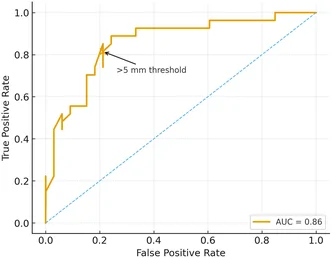
ROC curve depicting the diagnostic performance of arthroscopic LTP translation for identifying high‐grade pivot‐shift (grade ≥ 2). The AUC is 0.85 (95% CI, 0.77‐0.93), indicating excellent discriminative capacity. The operating point corresponding to a 5‐mm threshold (filled circle) provides 82% sensitivity and 78% specificity for detecting high‐grade rotational instability. The dashed diagonal line represents the reference line of no discrimination (AUC = 0.50). (AUC, area under the curve; CI, confidence interval; LTP, lateral tibial plateau; ROC, receiver operating characteristic.)

## DISCUSSION

This study shows that arthroscopic measurement of LTP translation obtained from standardized arthroscopic recordings is reproducible and is associated with clinical pivot‐shift severity and LTS, with a threshold showing useful discrimination for high‐grade pivot‐shift.^5^, ^7^, ^8^, ^9^, ^10^ These findings support the concept that visually observed anterolateral tibial plateau motion during arthroscopy reflects clinically relevant rotational laxity.^5^, ^6^, ^20^, ^21^, ^22^, ^23^, ^24^

Previous biomechanical and imaging studies have extensively explored the mechanisms of rotational instability and pivot‐shift assessment, underscoring the multifactorial nature of this phenomenon and the absence of a universally accepted reference standard.^7^, ^9^, ^25^ Clinical evaluation of pivot‐shift has traditionally relied on subjective grading, whereas more recent objective approaches have incorporated inertial sensors, navigation systems, and robotic or electromagnetic tracking technologies.^7^, ^8^ Although these methods have improved the precision of rotational analysis, their application remains largely confined to research settings and is not routinely available during standard ACL reconstruction.^7^, ^8^, ^9^, ^10^, ^25^, ^26^, ^27^, ^28^

In the present study, arthroscopic translation was initially analyzed as a continuous variable, showing a monotonic relation with pivot‐shift grade across the spectrum of instability. Dichotomization was subsequently performed to facilitate diagnostic analysis and clinical interpretability, consistent with common thresholds used to define clinically meaningful rotational instability. This approach allowed evaluation of discriminative performance while preserving the underlying biological plausibility of the measurement.

Importantly, the reported reproducibility reflects agreement in measurements derived from standardized arthroscopic video recordings rather than independent real‐time intraoperative measurements performed by different surgeons. Consequently, reliability metrics should be interpreted as consistency of measurement interpretation rather than variability of the intraoperative examination itself.

The current findings are consistent with prior biomechanical observations reported by LaPrade et al. and Monaco et al., who revealed that residual pivot‐shift after ACL reconstruction is influenced by both soft‐tissue and osseous factors.^22^, ^29^, ^30^ Cadaveric studies have highlighted the contribution of secondary stabilizers, including the anterolateral complex and meniscotibial attachments, whereas clinical investigations have shown associations between persistent pivot‐shift, posterior tibial slope, and lateral meniscal integrity.^28^, ^31^, ^32^ The present study aligns with these concepts by quantifying arthroscopically observed tibial plateau displacement under rotational stress.^16^, ^17^, ^25^, ^28^, ^31^, ^33^ Unlike traditional qualitative arthroscopic descriptions, the approach described here allows surgeons to express lateral tibial motion numerically using readily available instrumentation.^7^, ^8^

Although a calibration grid was used during the study to verify optical scaling of the arthroscopic system, routine application of the measurement does not require a dedicated grid. The calibrated probe markings themselves provide the reference for quantification, supporting the practicality of the technique in standard arthroscopic settings.

From a clinical perspective, quantitative documentation of arthroscopic LTP translation may assist surgeons in contextualizing rotational instability during ACL reconstruction.^34^, ^35^, ^36^, ^37^ This may help bridge the gap between subjective intraoperative perception and objective documentation of rotational laxity, without reliance on specialized sensors or navigation systems.^16^, ^17^, ^31^, ^33^, ^38^, ^39^

A translation threshold showing discriminative ability for high‐grade pivot‐shift may inform consideration of adjunctive anterolateral procedures, such as lateral extra‐articular tenodesis or anterolateral ligament reconstruction, within the broader clinical and anatomical context of each patient.^29^, ^30^, ^38^, ^39^ Additionally, standardized numerical reporting may facilitate communication across studies and support future research aimed at correlating intraoperative findings with postoperative outcomes.^29^, ^30^, ^34^, ^35^, ^38^, ^39^ The association between arthroscopic translation and rotational instability was consistent across sexes, suggesting applicability of the measurement in both male and female patients, although the study was not powered to define sex‐specific thresholds.

### Limitations

Several limitations of this study should be acknowledged. First, assessment of pivot‐shift severity remains constrained by the absence of a universally accepted gold standard.

Second, arthroscopic LTP translation was compared with the preoperative clinical pivot‐shift examination performed in awake patients. Although this reflects routine clinical practice, it is inherently subjective and less reliable than examination under anesthesia or instrumented assessment. The lack of intraoperative pivot‐shift testing or objective comparator limits validation of the proposed measurement.

Third, reliability analyses evaluated reproducibility of measurements obtained from standardized arthroscopic video recordings rather than independent real‐time intraoperative measurements by different surgeons. As such, the reported ICC values reflect consistency of interpretation of recorded displacement rather than true intersurgeon measurement variability. Independent intraoperative measurements were not feasible within the constraints of the operative workflow.

Fourth, no formal a priori sample size calculation or power analysis was performed before study initiation. Therefore, the sample size was determined by the number of eligible patients available during the study period, which may limit the precision of the estimates and increase the risk of type II error.

Finally, no direct comparison was performed with instrumented systems or navigation‐based assessments that could provide external validation. Postoperative outcomes were not systematically evaluated beyond early follow‐up, and therefore, the prognostic value of arthroscopic translation for long‐term rotational control cannot be determined from the present data.^32^, ^37^, ^40^

## CONCLUSIONS

Arthroscopic quantification of LTP translation is a reproducible and clinically relevant method for objectively assessing rotational instability in ACL‐deficient knees. Arthroscopic quantification of LTP translation showed excellent inter‐ and intraobserver reliability and was strongly associated with preoperative pivot‐shift grade. A translation threshold greater than 5 mm showed good discriminative performance for identifying high‐grade pivot‐shift.

## DISCLOSURES

The authors (H.R., C.H., C.C., M.P., M.M., C.P‐C., A.B.C., B.R., F.E.U.) declare that they have no known competing financial interests or personal relationships that could have appeared to influence the work reported in this article.
